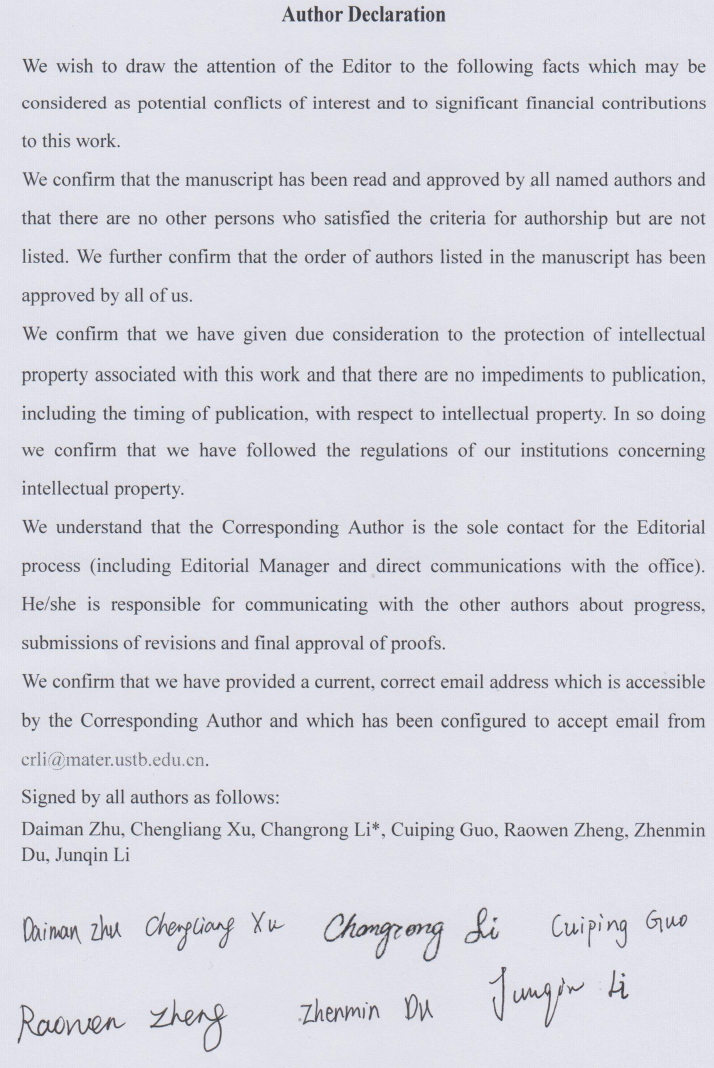# Data and analyses of phase relations in the Ce-Fe-Sb ternary system

**DOI:** 10.1016/j.dib.2017.10.075

**Published:** 2017-11-04

**Authors:** Daiman Zhu, Chengliang Xu, Changrong Li, Cuiping Guo, Raowen Zheng, Zhenmin Du, Junqin Li

**Affiliations:** a School of Materials Science and Engineering, University of Science and Technology Beijing, Beijing 100083, China; b Shenzhen Key Laboratory of Special Functional Materials, Shenzhen University, Shenzhen 518060, China

## Abstract

These data and analyses support the research article “Experimental study on phase relations in the Ce-Fe-Sb ternary system” Zhu et al. (2017) [1]. The data and analyses presented here include the experimental results of XRD, SEM and EPMA for the determination of the whole liquidus projection and the isothermal section at 823 K in the Ce-Fe-Sb system. All the results enable the understanding of the constituent phases and the solidification processes of the as-cast alloys as well as the phase relations and the equilibrium regions at 823 K in the Ce-Fe-Sb ternary system over the entire composition.

**Specifications Table**

**Table:** 

Subject area	Materials Sciences
**More specific subject area**	*Phase diagram and relations*
**Type of data**	*Tables, figures, images (x-ray, microscopy, etc.),*
**How data was acquired**	*Microscope, SEM, XRD, EPMA of as-cast and annealed samples*
**Data format**	*Raw, analyzed*
**Experimental factors**	*The as-cast samples were prepared and the isothermal heat treatment was conducted as reported in a previous study**[1]*.
**Experimental features**	*The as-cast alloys were used to determine the primarily crystallized phases, to construct the liquidus projection diagram, and to analyze the solidification processes. And the heat-treated samples were prepared to measure the phase equilibria and to construct the isothermal section.*
**Data source location**	*Data was collected in the School of Materials Science and Engineering, University of Science and Technology Beijing, Beijing 100083, China. (39°26N, 117°30 E)*
**Data accessibility**	*The data are available with this article and within the Ref.**[1]*
**Related research article**	*Experimental study on phase relations in the Ce-Fe-Sb ternary system (in press)*

**Value of the Data**

(1) These data presented for the measurement of the liquidus projections in the Ce-Fe-Sb system have never been made public before.
(2) These data create understanding of the phase relations and phase equilibria of the Ce-Fe-Sb ternary in detail.
(3) Future thermodynamic assessments of the Ce-Fe-Sb ternary or related systems can be based on these data to construct a multi-component thermodynamical database.

## 1 Data

These raw data, containing the experimental results of XRD, SEM and EPMA for the determination of the whole liquidus projection and the isothermal section at 823 K in the Ce-Fe-Sb system, are obtained from Ref. [1].

## 2 Experimental design, materials, and methods

All experimental design, materials and methods were based on reported paper [1].

### 2.1 Liquidus projection over the entire composition range

For the liquidus projection of the Ce-Fe-Sb ternary system constructed in present work, there exist 17 primary solidification regions. Except the descriptions of the primary solidification regions (CeSb), (FeSb), τ_1_, τ_2_ and τ_3_ in Ref. [1], the remaining regions are fully shown and described as follows.

It should be noted that within the XRD spectra of the as-cast alloys #1–10, some diffraction peaks of Ce_2_O_3_ exist since the very easy oxidation of these Ce-rich alloys after their exposing to air, resulting in the dark-grey distributions of cracks and pits along with the dark phase (γCe) within the backscattered electron images.

#### 2.1.1 Primary solidification region of (α/δFe)

1) Ce-72Fe-25Sb (#1)

**Fig. 1:**
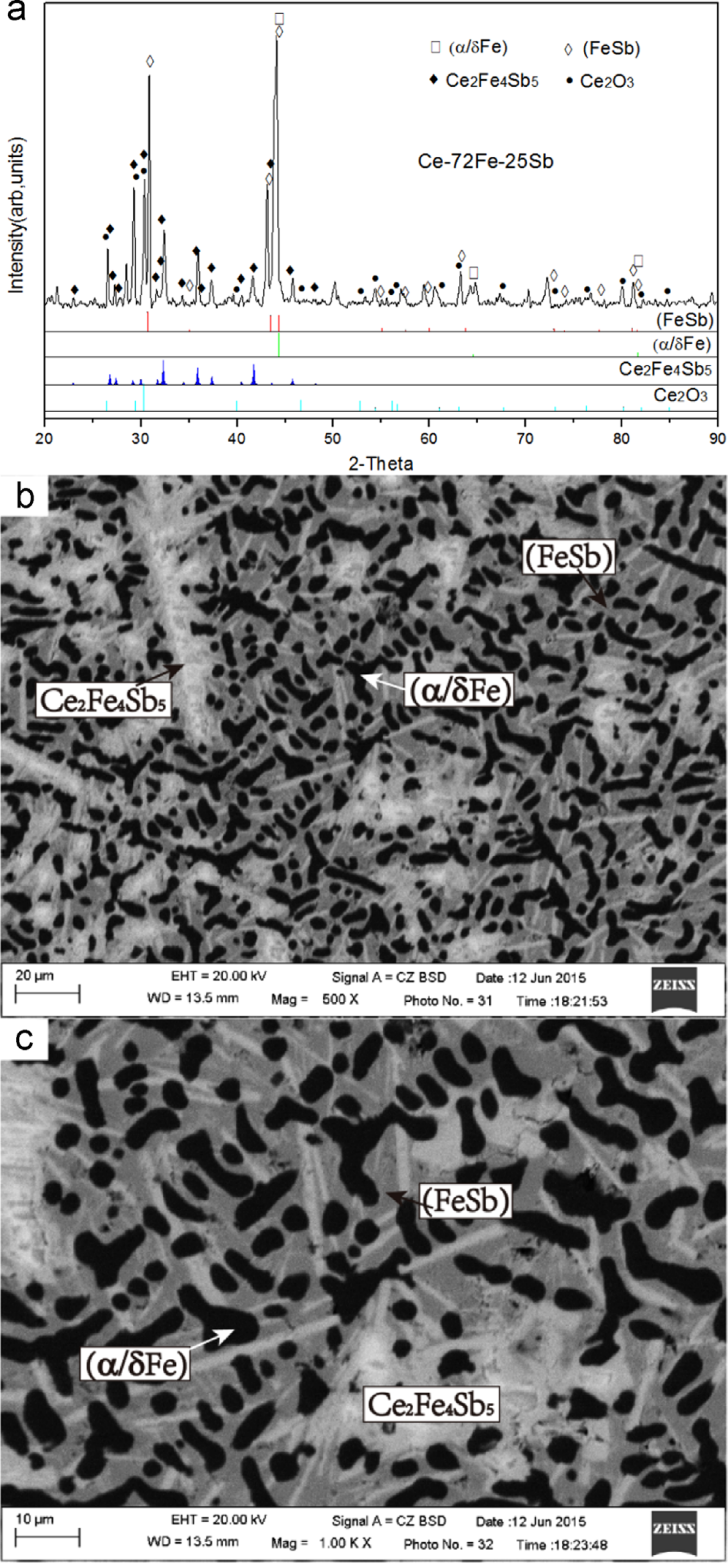
Experimental results of Ce-72Fe-25Sb (#1) as-cast alloy: (a) X-ray diffractogram; (b) and (c) BEI micrographs at low and high magnifications, respectively.

The X-ray diffractogram and BEI micrographs of Ce-72Fe-25Sb (#1) as-cast alloy are shown in Fig. 1. The microstructure consists of a large and black primary phase (α/δFe), a dark+grey two-phase eutectic (α/δFe)+(FeSb) divorced to some degree, and a grey+white two-phase eutectic (FeSb)+τ_3_ which was also divorced. The crystal structure and the composition of the different phases were determined using XRD and EPMA respectively. Within the XRD spectra of the as-cast samples as shown in Fig. 1(a), some diffraction picks of Ce_2_O_3_ exist since the very easy oxidation of the Ce-containing alloys after their exposing to air. The average compositions of the primary solidification phase (α/δFe) and the subsequent solidification phases (FeSb) and τ_3_ in the divorced eutectic structures are Ce-96.97Fe-3.00Sb, Ce-54.21Fe-44.00Sb and Ce-36.86Fe-44.97Sb respectively as listed in Table 1.

2) Ce-60Fe-33Sb (#2)

**Table 1:** The compositions of the constituent phases existing in the different Ce-Fe-Sb ternary as-cast alloys.

Phases	Compositions (at%)			Remarks
	Ce	Fe	Sb	
(α/δFe)	0.03	96.97	3.00	In as-cast #1 alloy
	0.07	98.35	1.58	In as-cast #2 alloy
	0.16	96.24	3.60	In as-cast #11 alloy
	0.98	96.33	2.69	In as-cast #12 alloy
	0.00	95.65	4.35	In as-cast #24 alloy
	0.11	96.18	3.71	In as-cast #25 alloy
	0.13	95.98	3.89	In as-cast #26 alloy
				
(γFe)	0.39	98.14	1.47	In as-cast #3 alloy
Ce_2_Fe_17_	12.99	87.01	0.00	In as-cast #3 alloy
	9.55	90.45	0.00	In as-cast #4 alloy
	10.19	89.81	0.00	In as-cast #5 alloy
	10.89	89.11	0.00	In as-cast #9 alloy
	9.59	90.41	0.00	In as-cast #10 alloy
	11.49	88.51	0.00	In as-cast #25 alloy
	10.57	89.43	0.00	In as-cast #26 alloy
				
CeFe_2_	32.99	67.01	0.00	In as-cast #3 alloy
	32.20	67.80	0.00	In as-cast #4 alloy
	32.32	67.68	0.00	In as-cast #5 alloy
	66.08	33.92	0.00	In as-cast #6 alloy
	33.66	66.34	0.00	In as-cast #7 alloy
	33.44	66.51	0.00	In as-cast #8 alloy
				
(γCe)	98.41	0.00	1.59	In as-cast #6 alloy
	98.60	0.00	1.40	In as-cast #7 alloy
	96.68	2.25	1.07	In as-cast #8 alloy
				
(δCe)	97.90	0.75	1.35	Derived
Ce_2_Sb	66.92	0.00	33.08	In as-cast #3 alloy
	66.94	0.00	33.16	In as-cast #4 alloy
	67.89	0.00	32.10	In as-cast #5 alloy
	66.94	0.00	33.06	In as-cast #6 alloy
	66.78	0.00	33.22	In as-cast #7 alloy
	66.05	0.00	33.95	In as-cast #8 alloy
	66.70	0.00	33.30	In as-cast #9 alloy
	66.79	0.00	33.21	In as-cast #10 alloy
				
Ce_4_Sb_3_	55.90	0.00	44.10	In as-cast #9 alloy
	54.44	0.00	45.56	In as-cast #10 alloy
				
(CeSb)	49.36	0.00	50.64	In as-cast #11 alloy
	49.68	1.11	49.21	In as-cast #12 alloy
	49.66	0.00	50.34	In as-cast #13 alloy
	49.47	0.00	50.52	In as-cast #14 alloy
	49.82	0.10	50.07	In as-cast #15 alloy
	48.73	0.75	50.52	In as-cast #16 alloy
	48.50	1.61	49.88	In as-cast #17 alloy
	48.71	1.61	49.68	In as-cast #25 alloy
	48.62	1.59	49.79	In as-cast #26 alloy
				
βCeSb_2_	33.42	0.12	66.46	Derived
αCeSb_2_	33.96	0.00	66.04	In as-cast #18 alloy
	33.16	0.00	66.84	In as-cast #19 alloy
	33.50	0.69	66.50	In as-cast #20 alloy
	33.94	0.00	66.06	In as-cast #21 alloy
	33.03	0.00	66.97	In as-cast #22 alloy
				
(Sb)	0.00	0.04	99.96	In as-cast #18 alloy
	0.71	0.83	98.46	In as-cast #19 alloy
	0.21	0.00	99.79	In as-cast #20 alloy
	0.00	0.10	99.90	In as-cast #21alloy
	0.00	0.08	99.92	In as-cast #22alloy
				
FeSb_2_	33.52	0.00	66.48	Derived
(FeSb)	1.78	54.21	44.00	In as-cast #1 alloy
	1.90	52.01	46.09	In as-cast #13 alloy
	1.89	55.03	43.08	In as-cast #14 alloy
	1.11	52.3	46.59	In as-cast #16 alloy
	0.08	54.44	45.48	In as-cast #17 alloy
	0.73	52.72	46.55	In as-cast #19 alloy
	1.00	55.20	43.80	In as-cast #20alloy
	0.14	51.23	48.63	In as-cast #21 alloy
	0.16	50.54	49.40	In as-cast #23 alloy
	1.09	58.32	40.60	In as-cast #24alloy
				
CeFe_4_Sb_12_(τ_1_)	4.52	25.10	70.38	In as-cast #18 alloy
	5.12	25.31	69.57	In as-cast #19 alloy
	4.27	26.00	69.73	In as-cast #20 alloy
	5.12	23.13	69.75	In as-cast #21 alloy
	5.91	24.06	70.03	In as-cast #22 alloy
	5.48	25.04	69.48	In as-cast #23 alloy
				
CeFeSb_2_(τ_2_)	28.61	24.94	46.45	In as-cast #11 alloy
	26.47	24.22	49.31	In as-cast #12 alloy
	28.22	25.34	46.43	In as-cast #13 alloy
	27.77	24.52	47.71	In as-cast #23 alloy
				
Ce_2_Fe_4_Sb_5_(τ_3_)	18.17	36.86	44.97	In as-cast #1 alloy
	17.23	35.89	46.88	In as-cast #2 alloy
	17.53	38.17	44.30	In as-cast #11 alloy
	19.13	37.13	43.74	In as-cast #12 alloy
	18.14	36.83	45.02	In as-cast #15 alloy
	17.66	37.41	44.93	In as-cast #16 alloy
	17.86	36.56	45.58	In as-cast #17 alloy
	18.11	36.18	45.71	In as-cast #24 alloy

**Fig. 2:**
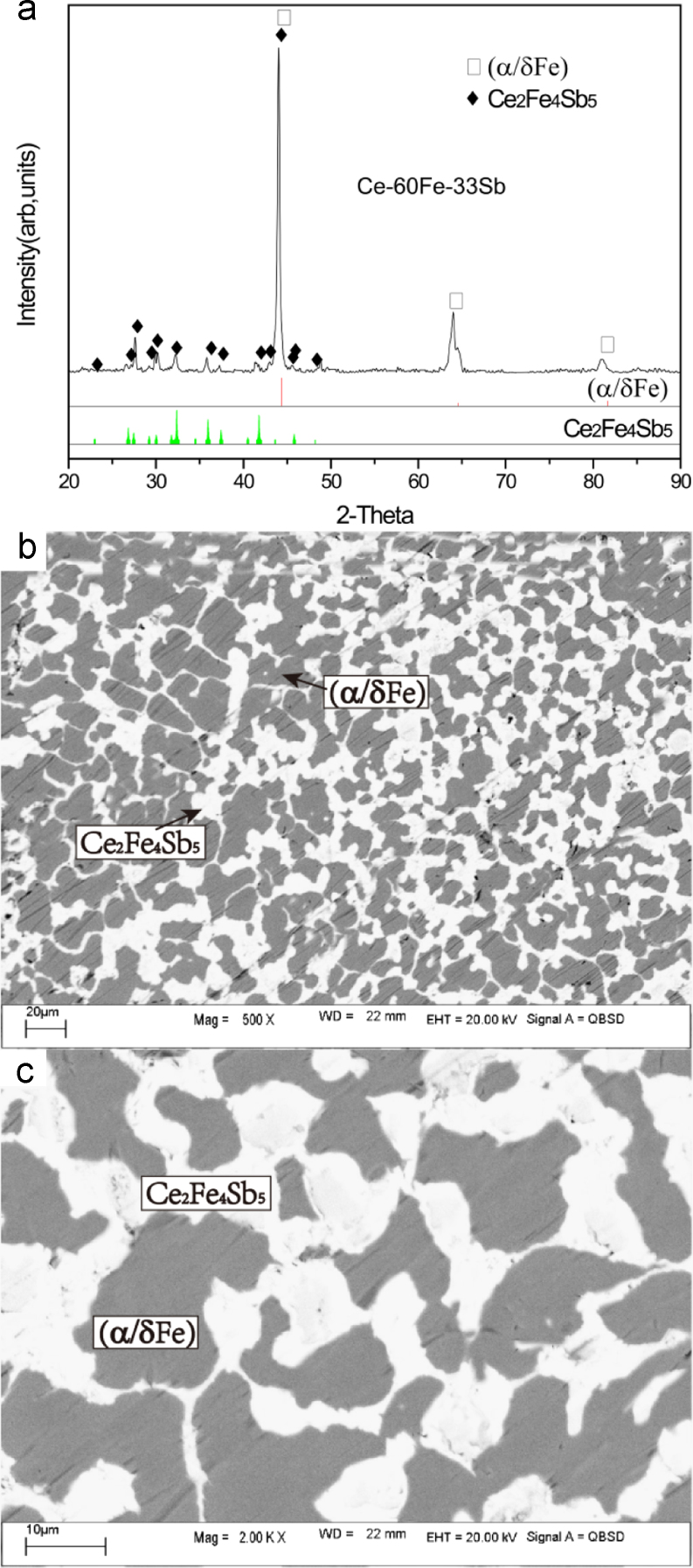
Experimental results of Ce-60Fe-33Sb (#2) as-cast alloy: (a) X-ray diffractogram; (b) and (c) BEI micrographs at low and high magnifications, respectively.

The microstructure of Ce-60Fe-33Sb (#2) as-cast alloy consists of a large and grey primary phase (α/δFe) with an average composition of Ce-98.35Fe-1.58Sb and an almost equal amount of white τ_3_ phase of Ce-35.89Fe-46.88Sb resulting in a divorced two-phase eutectic (α/δFe)+τ_3_ (Fig. 2).

**Fig. 3:**
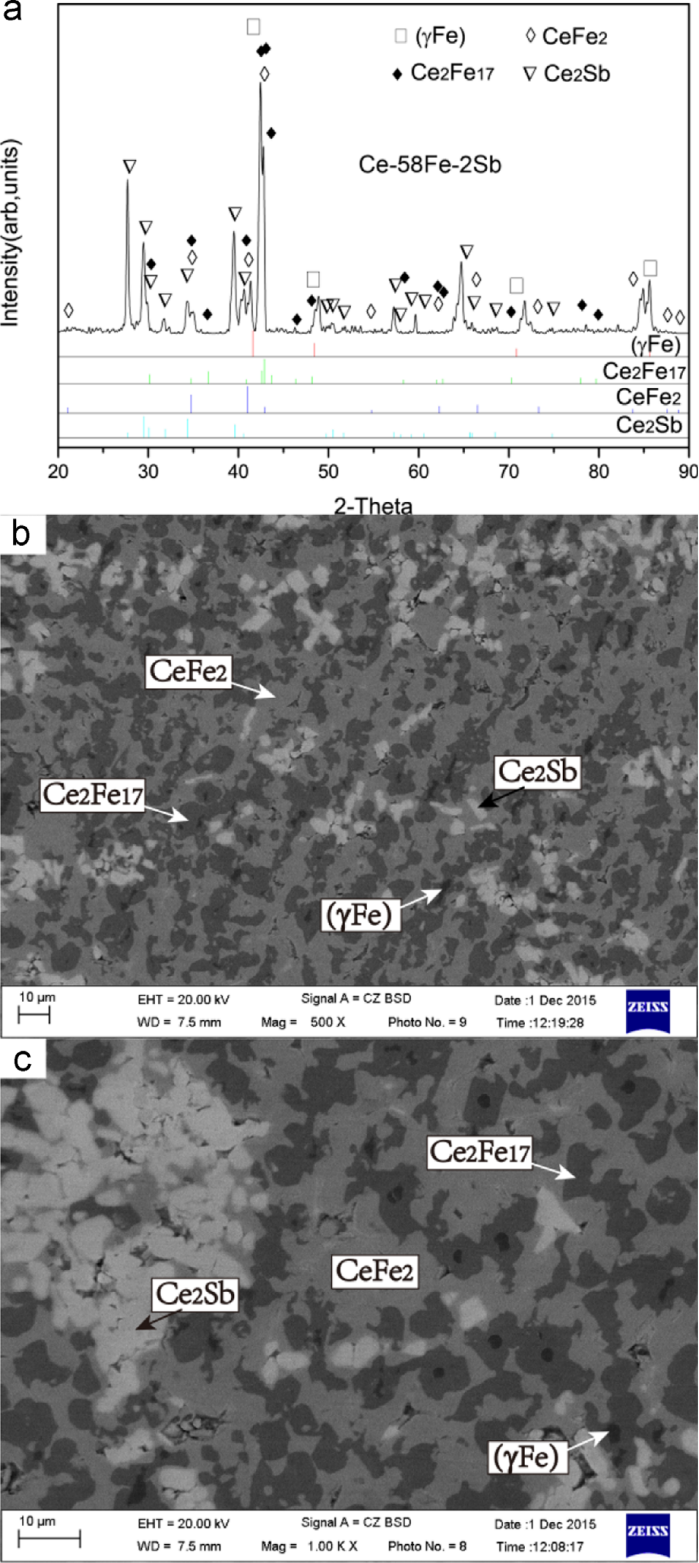
Experimental results of Ce-58Fe-2Sb (#3) as-cast alloy: (a) X-ray diffractogram; (b) and (c) BEI micrographs at low and high magnifications, respectively.

**Fig. 4:**
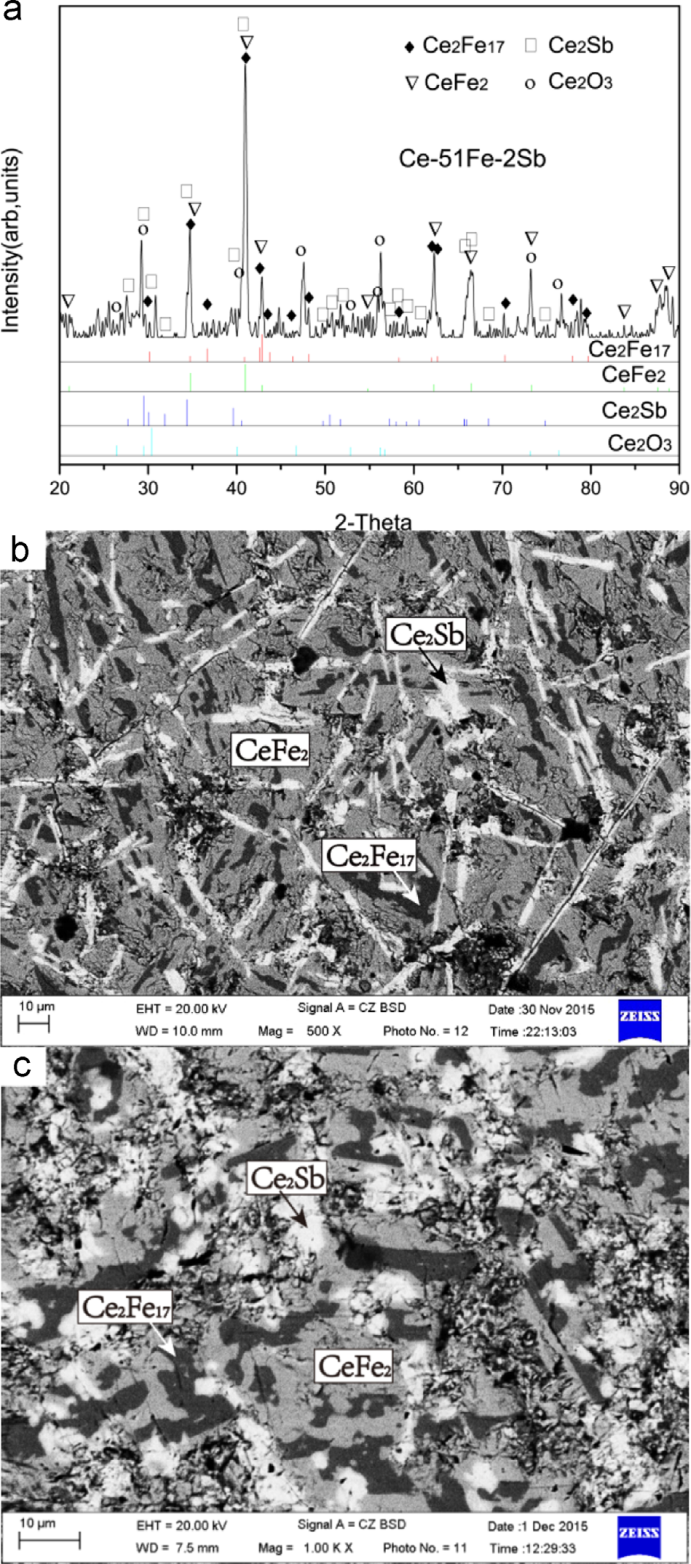
Experimental results of Ce-51Fe-2Sb (#4) and Ce-42Fe-6Sb (#5) as-cast alloys: (a) X-ray diffractogram of #4 alloy; (b) and (c) BEI micrographs of #4 and #5 alloys, respectively.

**Fig. 5:**
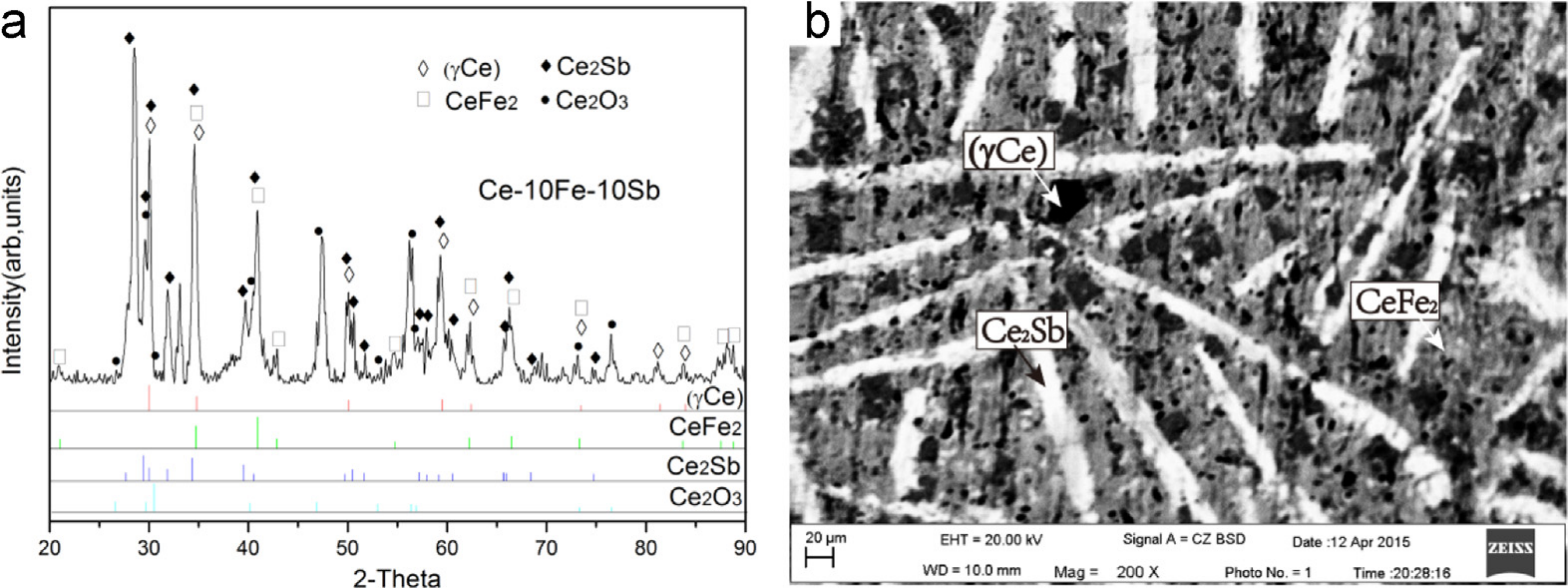
Experimental results of Ce-10Fe-10Sb (#6) as-cast alloy: (a) X-ray diffractogram; (b) BEI micrograph.

**Fig. 6:**
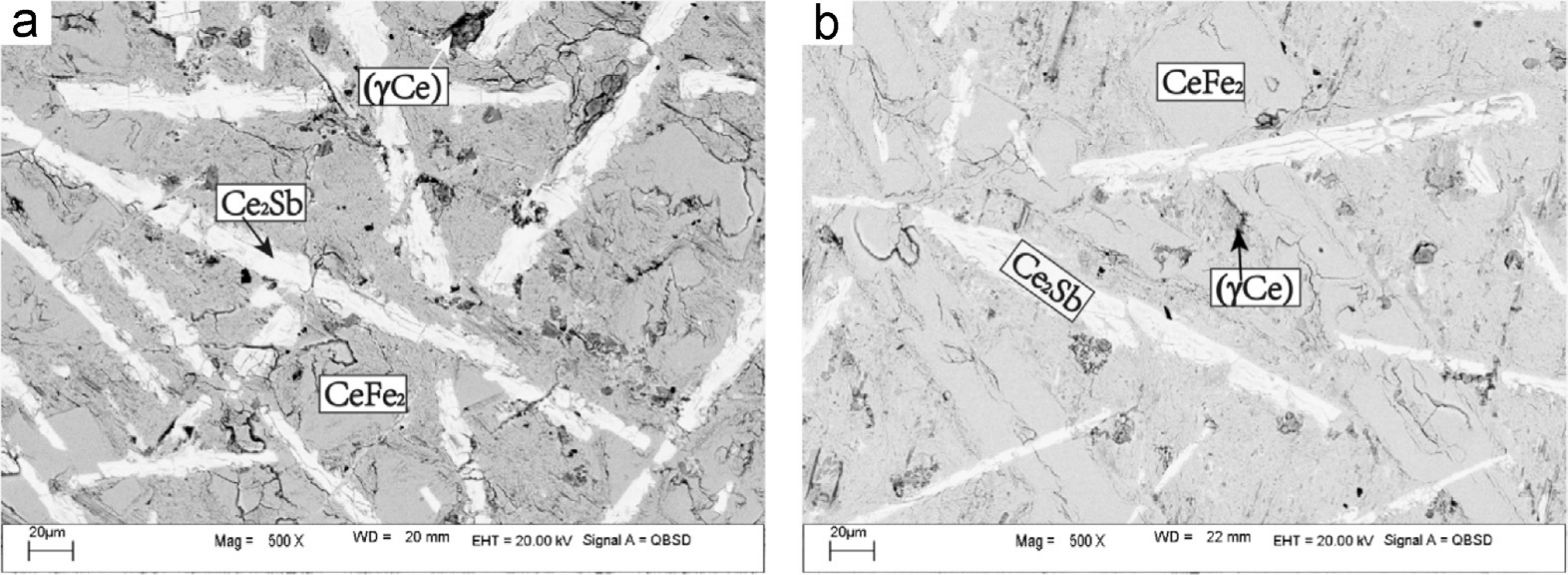
BEI micrographs of (a) Ce-22Fe-8Sb (#7) and (b) Ce-30Fe-10Sb (#8) as-cast alloys.

**Fig. 7:**
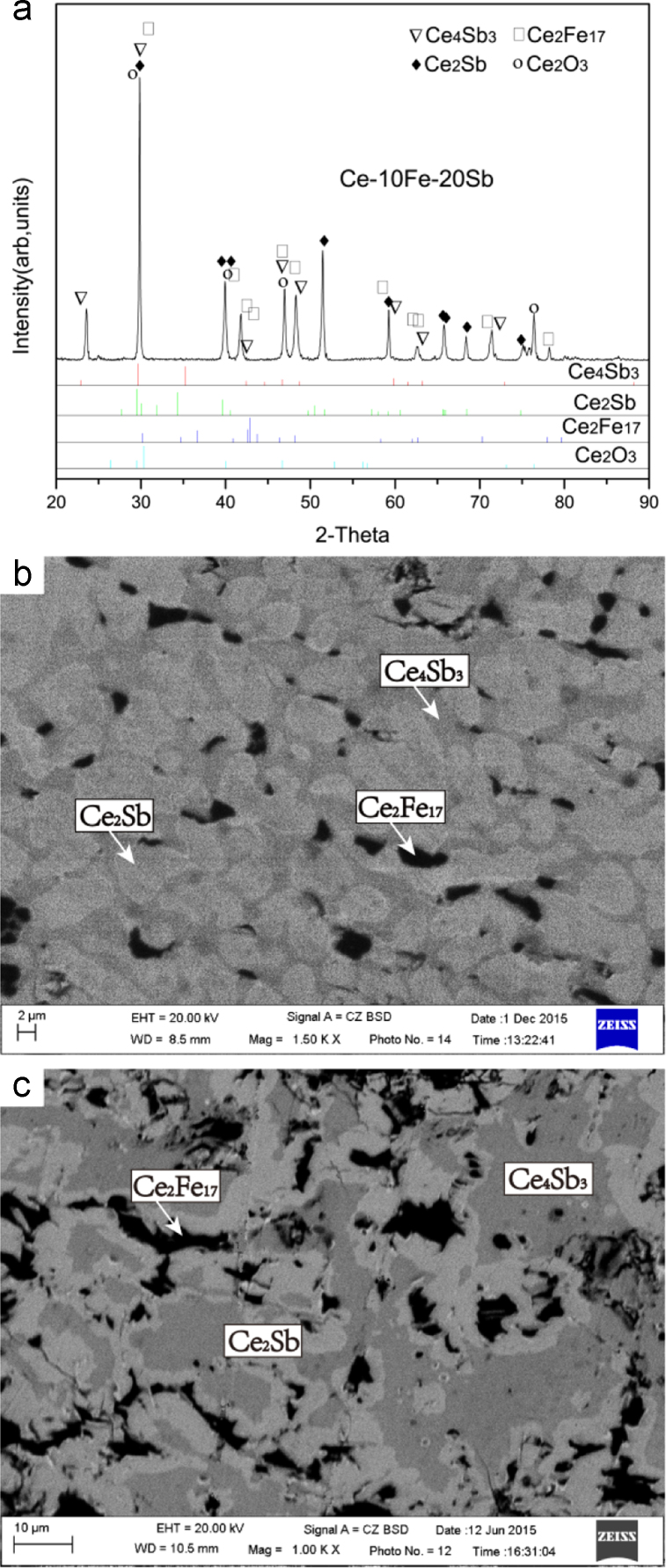
Experimental results of Ce-10Fe-20Sb (#9) and Ce-22Fe-16Sb (#10) as-cast alloys: (a) X-ray diffractogram of #9 alloy; (b) and (c) BEI micrographs of #9 and #10 alloys, respectively.

**Fig. 8:**
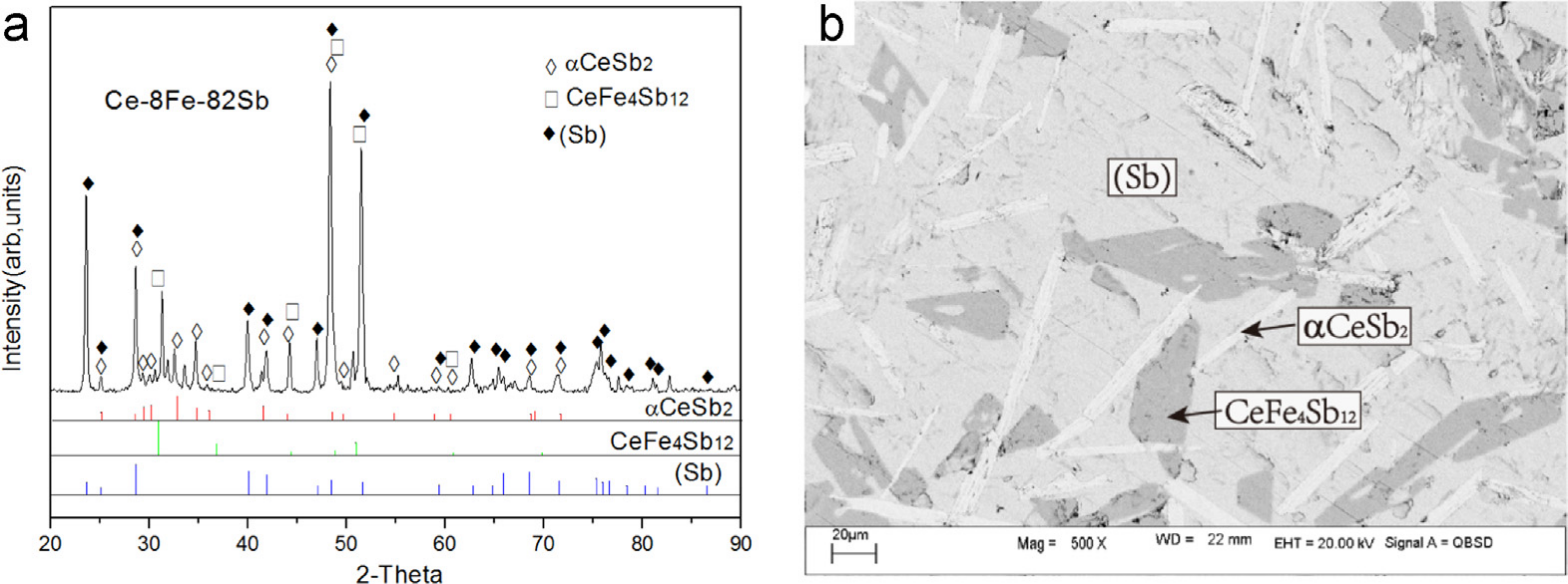
Experimental results of Ce-8Fe-82Sb (#18) as-cast alloy: (a) X-ray diffractogram; (b) BEI micrograph.

**Fig. 9:**
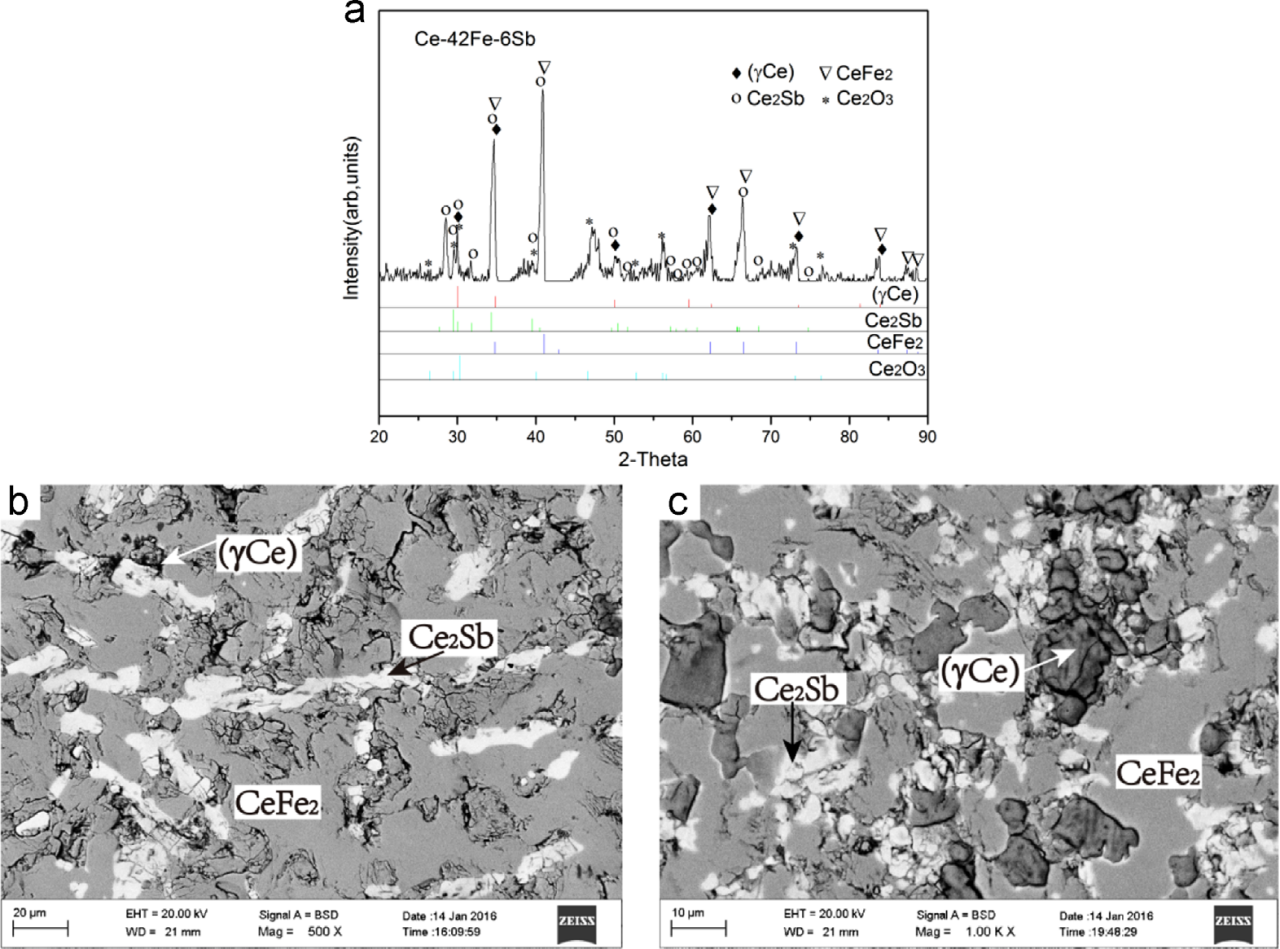
Isothermal experimental results of Ce-51Fe-2Sb (#4) and Ce-42Fe-6Sb (#5) heat-treated alloys: (a) X-ray diffractogram of #5 alloy; (b) and (c) BEI micrographs of #5 and #4 alloys, respectively.

**Fig. 10:**
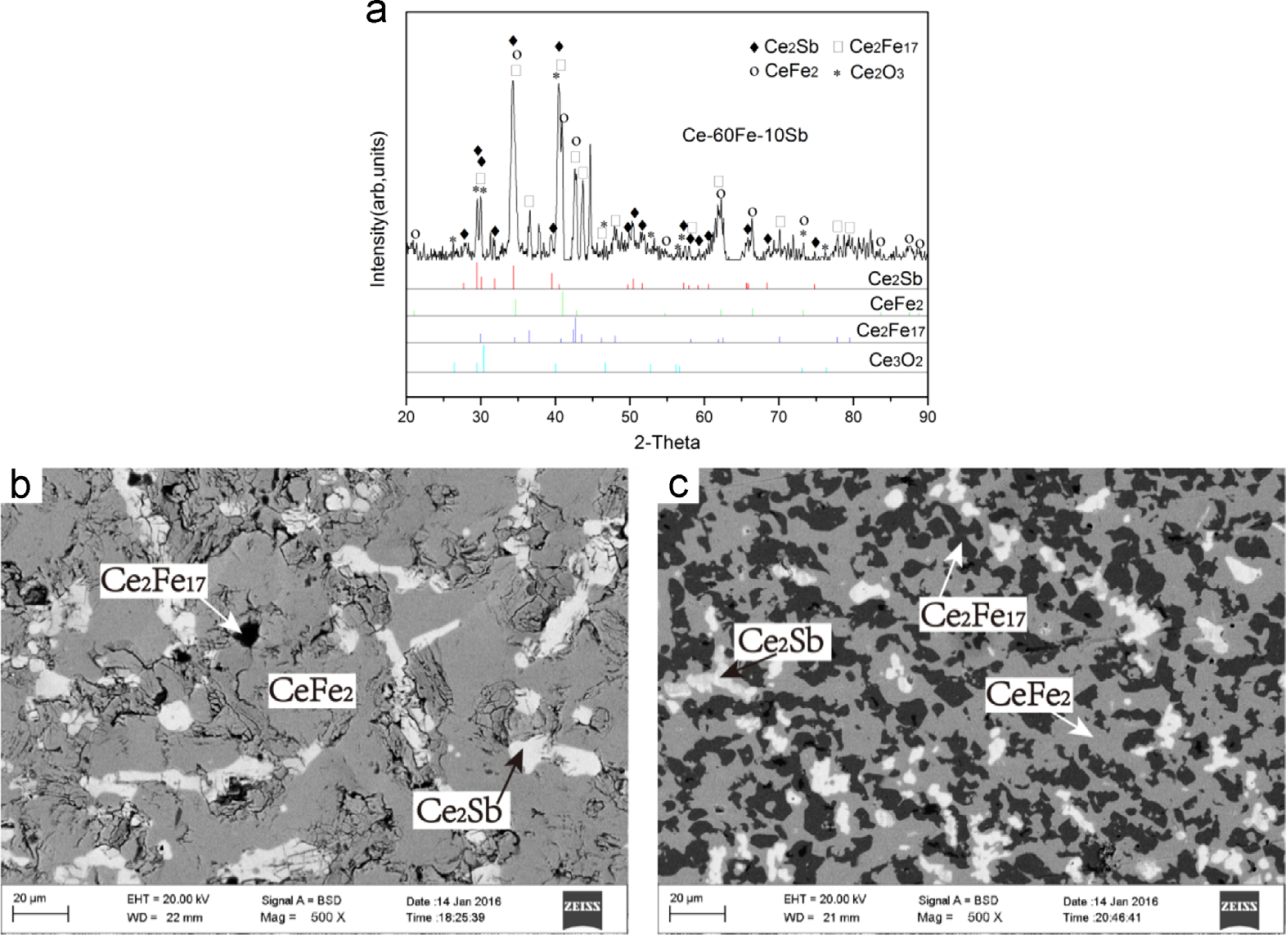
Isothermal experimental results of Ce-60Fe-10Sb (#27) and Ce-71Fe-3Sb (#28) heat-treated alloys: (a) X-ray diffractogram of #28 alloy; (b) and (c) BEI micrographs of #27 and #28 alloys respectively.

**Fig. 11:**
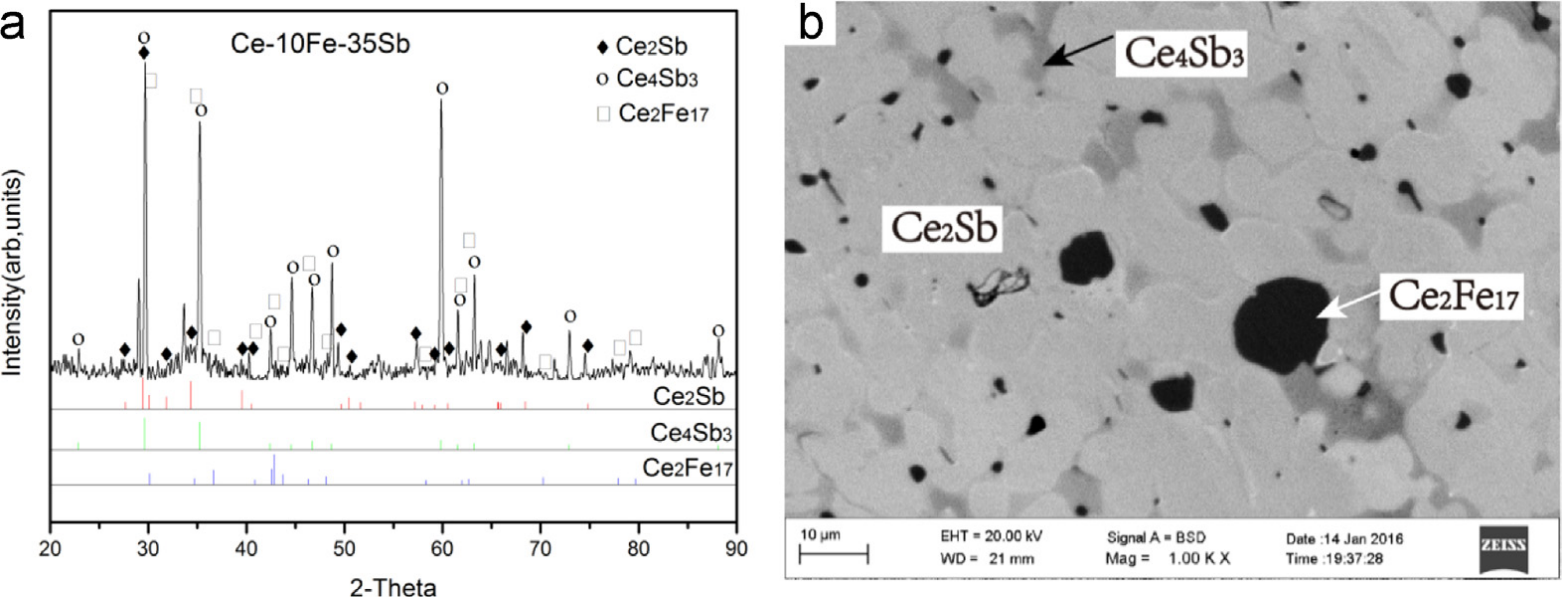
Isothermal experimental results of Ce-10Fe-35Sb (#29) heat-treated alloy:(a) X-ray diffractogram; (b) BEI micrograph.

**Fig. 12:**
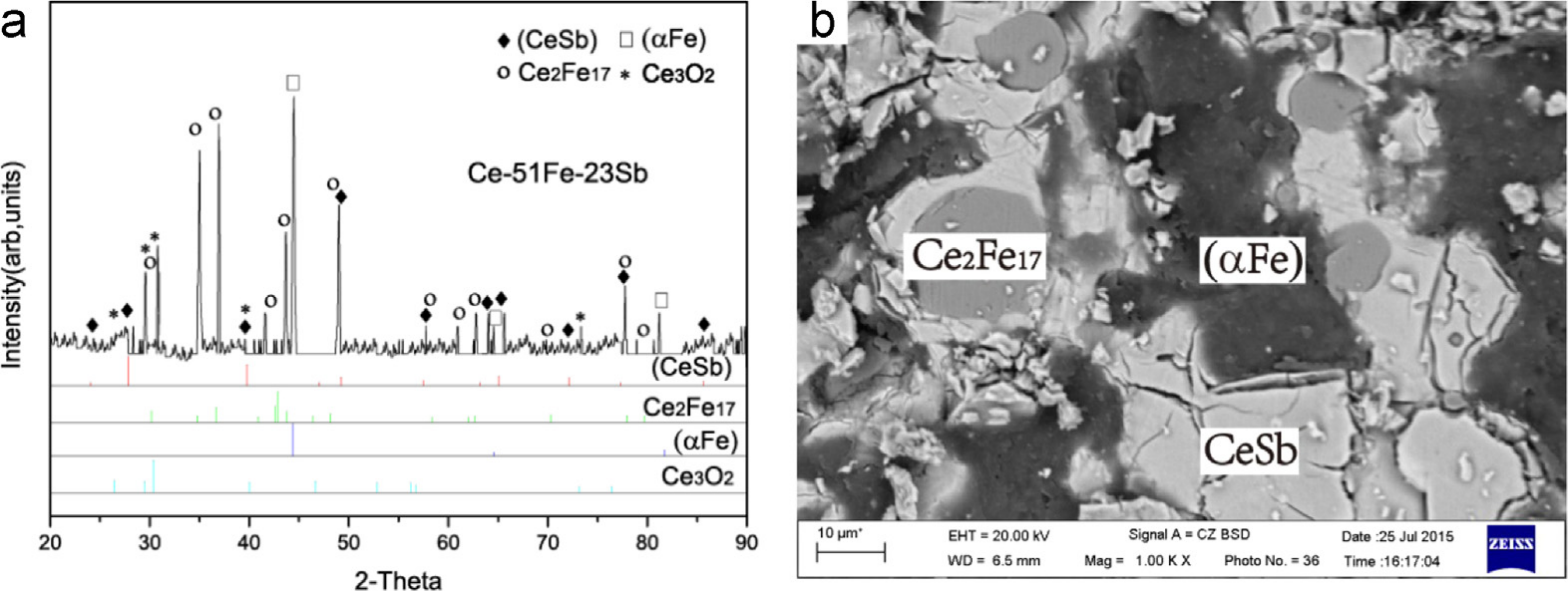
Isothermal experimental results of Ce-51Fe-23Sb (#30) heat-treated alloy:(a) X-ray diffractogram; (b) BEI micrograph.

**Fig. 13:**
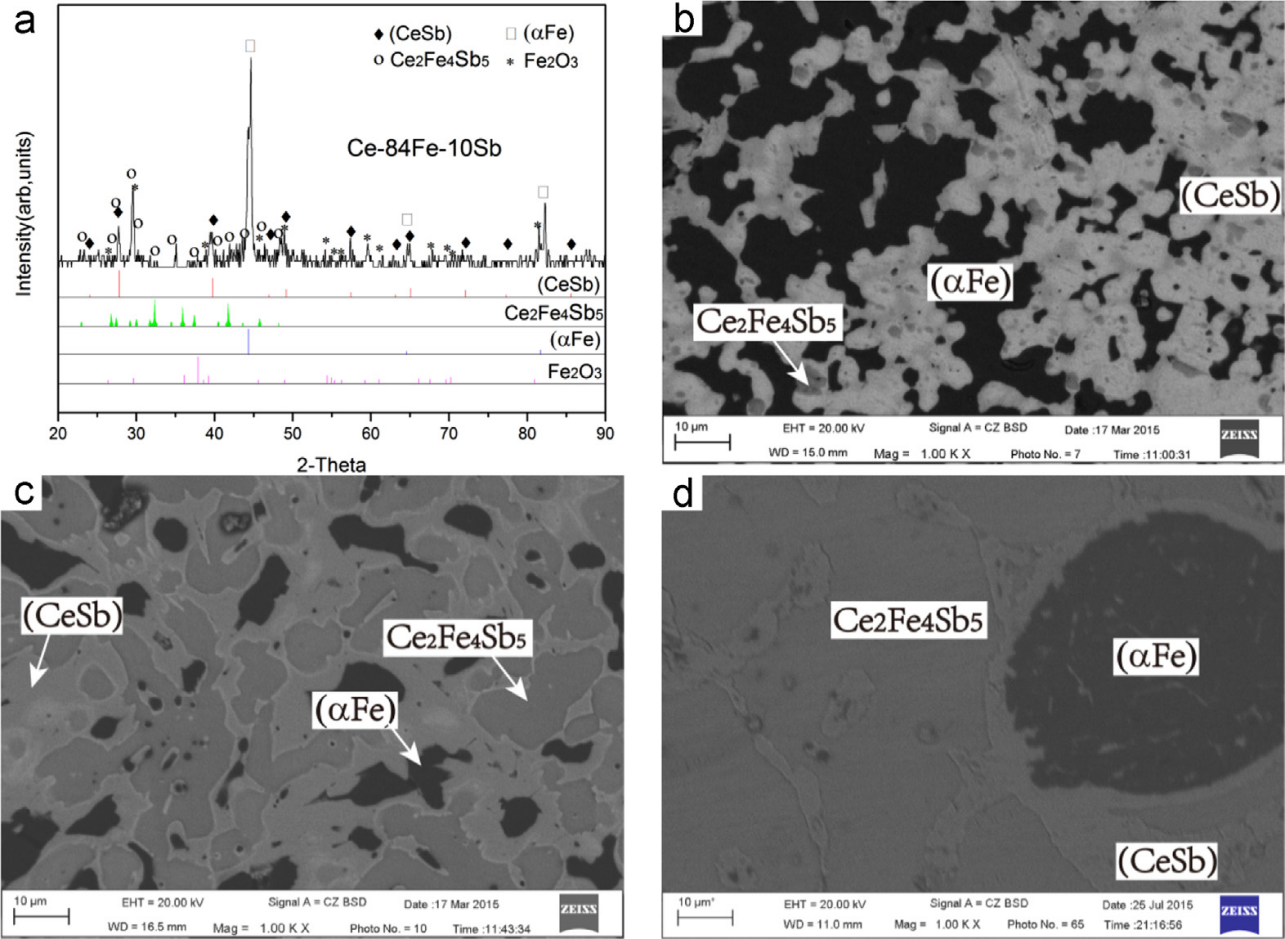
Isothermal experimental results of Ce-35Fe-45Sb(#11), Ce-16Fe-48Sb(#12) and Ce-84Fe-10Sb(#25) heat-treated alloys. (a) X-ray diffractogram of #26 alloy; (b), (c) and (d) BEI micrographs of #25, #11 and #12 alloys respectively.

**Fig. 14:**
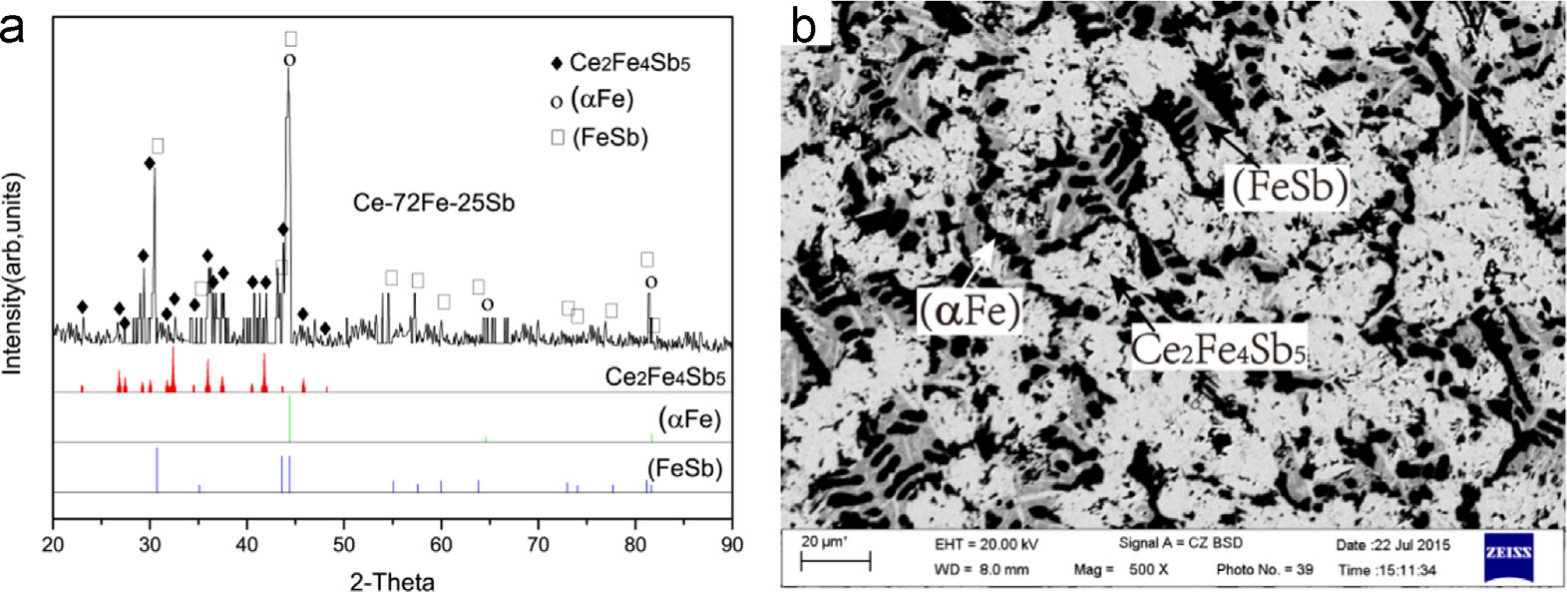
Isothermal experimental results of Ce-72Fe-25Sb (#1) heat-treated alloy:(a) X-ray diffractogram; (b) BEI micrograph.

**Fig. 15:**
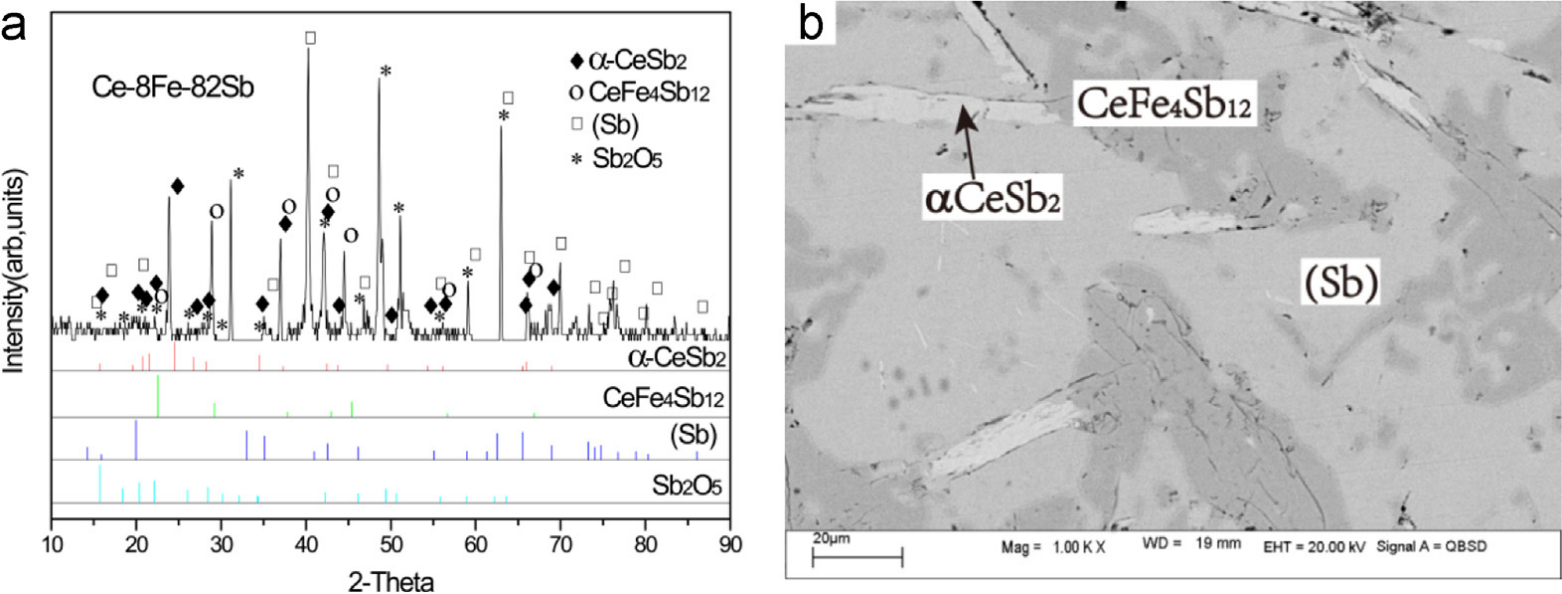
Isothermal experimental results of Ce-8Fe-82Sb (#14) heat-treated alloy:(a) X-ray diffractogram; (b) BEI micrograph.

**Fig. 16:**
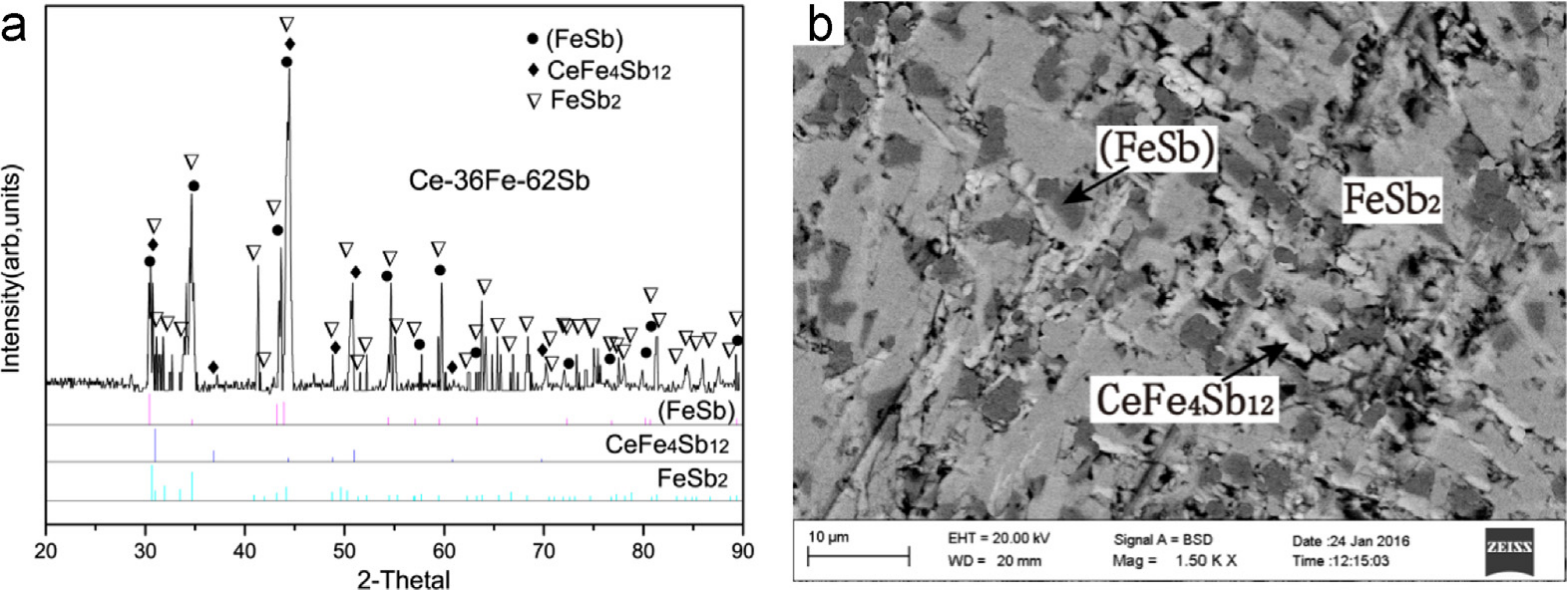
Isothermal experimental results of Ce-31Fe-65Sb (#19) heat-treated alloy:(a) X-ray diffractogram; (b) BEI micrograph.

**Fig. 17:**
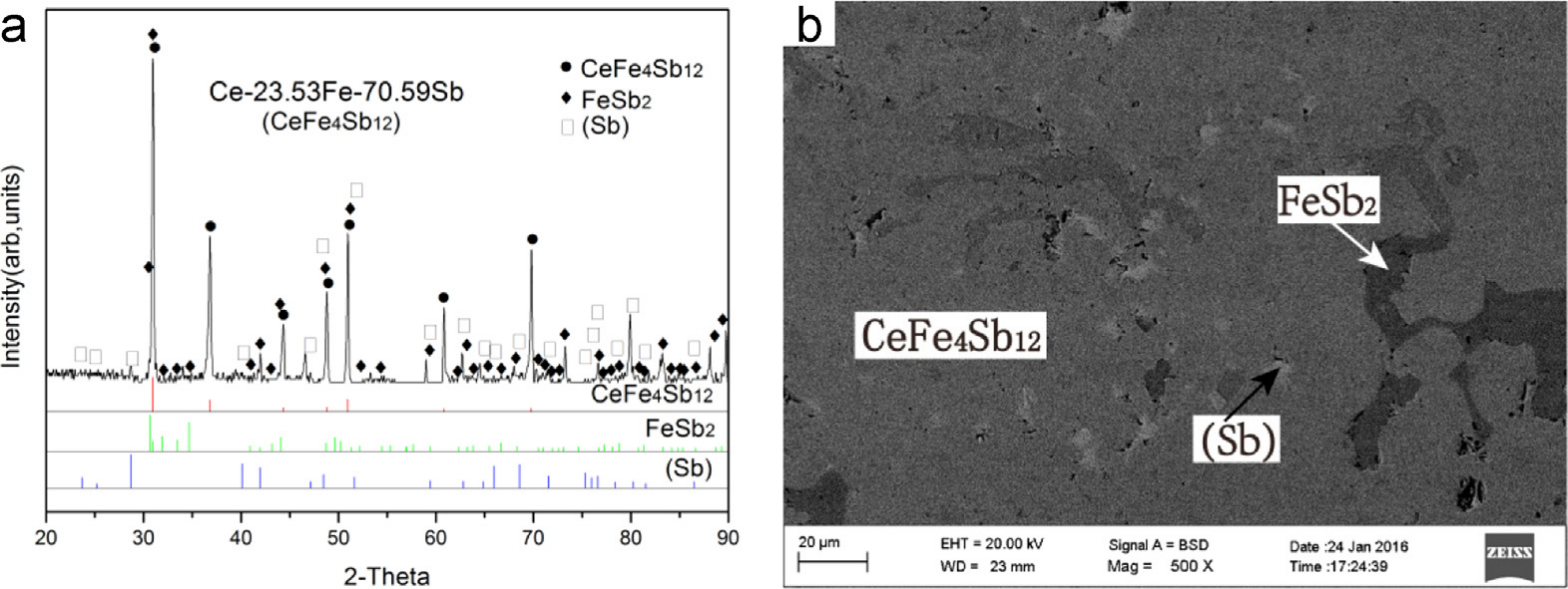
Experimental results of Ce-23.53Fe-70.59Sb (#21) heat-treated alloy:(a)X-ray diffractogram; (b) BEI micrograph.

**Fig. 18:**
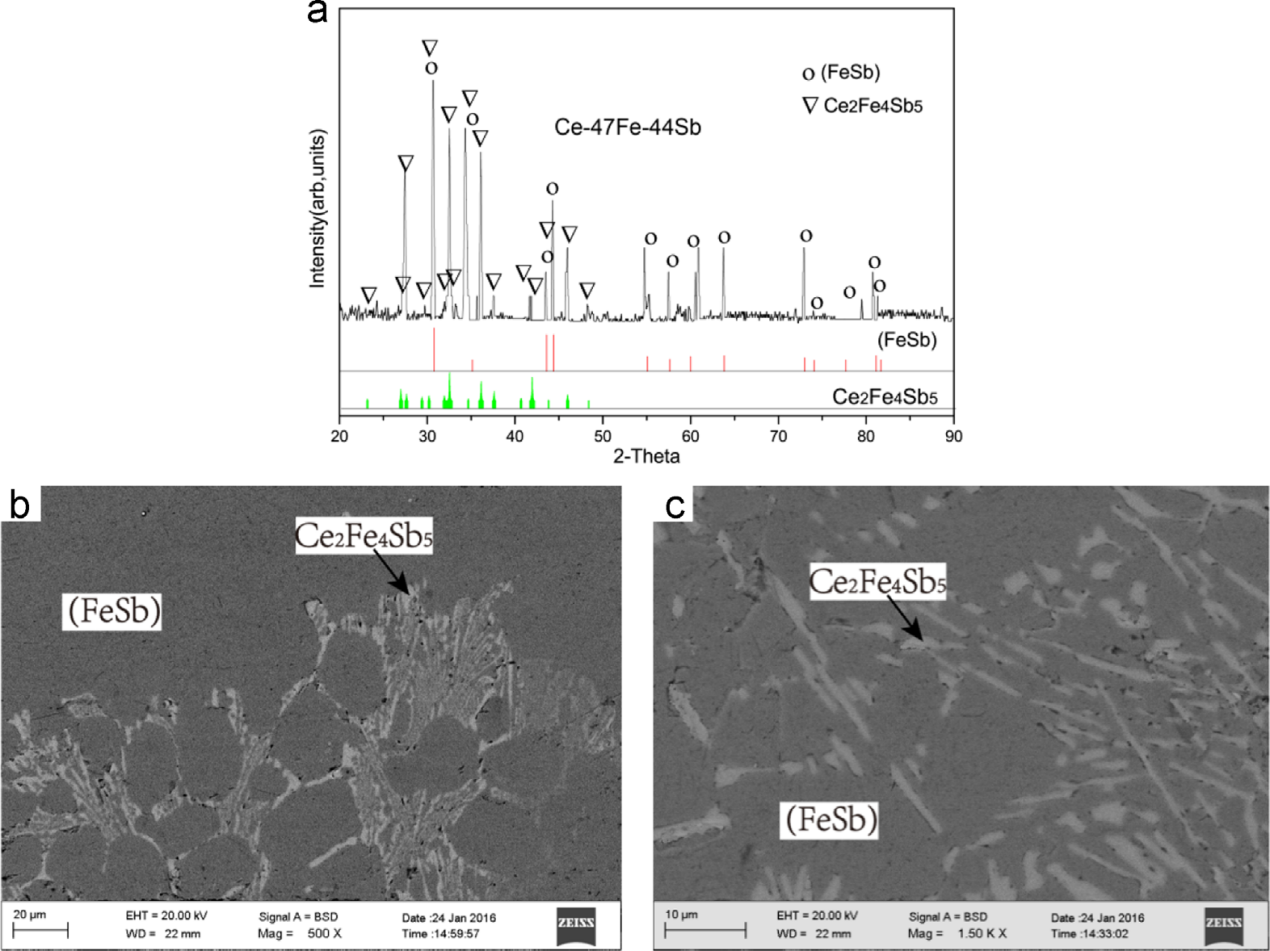
Isothermal experimental results of Ce-47Fe-44Sb (#24) heat-treated alloy: (a)X-ray diffractogram; (b) and (c) BEI micrographs at low and high magnifications, respectively.

#### 2.1.2 Primary solidification region of (γFe)

Ce-58Fe-2Sb (#3)

The microstructure of Ce-58Fe-2Sb (#3) as-cast alloy consists of a small and black primary phase (γFe), a dark-grey phase Ce_2_Fe_17_ which grew in a peritectic-type manner from the phase (γFe), a light-grey phase CeFe_2_ and a light-grey + white two-phase eutectic CeFe_2_ + Ce_2_Sb. The primary phase (γFe) is of a small volume fraction, indicating that the nominal composition of #3 as-cast alloy is very close to the peritectic ridge L+(γFe)→Ce_2_Fe_17_ (Fig.3).

#### 2.1.3 Primary solidification region of Ce_2_Fe_17_

Ce-51Fe-2Sb (#4) and Ce-42Fe-6Sb (#5)

The microstructures of Ce-51Fe-2Sb (#4) and Ce-42Fe-6Sb (#5) as-cast alloys consist of a dark flaky primary phase Ce_2_Fe_17_, a grey phase CeFe_2_ which grew in a peritectic manner from the phase Ce_2_Fe_17_, and a grey+white two-phase eutectic CeFe_2_+Ce_2_Sb (Fig.4).

#### 2.1.4 Primary solidification region of Ce_2_Sb

1) Ce-10Fe-10Sb (#6)

The microstructure of Ce-10Fe-10Sb (#6) as-cast alloy consists of a large and white dendrite primary phase Ce_2_Sb, a white+dark two-phase eutectic Ce_2_Sb+(γCe) and a white+dark+grey three-phase eutectic Ce_2_Sb+(γCe)+CeFe_2_ (Fig. 5).

2) Ce-22Fe-8Sb (#7) and Ce-30Fe-10Sb (#8)

Ce-22Fe-8Sb (#7) and Ce-30Fe-10Sb (#8) have the similar as-cast microstructure as shown in Fig. A6, consisting of a large and white dendrite primary phase Ce_2_Sb, a white+grey two-phase divorced eutectic Ce_2_Sb+CeFe_2_ and a white+grey+dark three-phases eutectic Ce_2_Sb+CeFe_2_+(γCe) which was also divorced (Fig. 6).

#### 2.1.5 Primary solidification region of Ce_4_Sb_3_

*Ce-10Fe-20Sb (#9) and Ce-22Fe-16Sb (#10)*

The microstructure of Ce-10Fe-20Sb (#9) and Ce-22Fe-16Sb (#10) as-cast alloys consists of a large dark grey primary phase Ce_4_Sb_3_ with an average composition of Ce-44.10Sb, a light grey Ce_2_Sb phase of Ce-33.30Sb growing in a peritectic-type manner, and a dark Ce_2_Fe_17_ phase of Ce-90.41Fe divorced from the two-phase eutectic Ce_2_Sb+Ce_2_Fe_17_ (Fig. 7).

#### 2.1.6 Primary solidification region of CeSb_2_

Ce-8Fe-82Sb (#18)

The microstructure Ce-8Fe-82Sb (#18) as-cast alloy consists of a white strip primary phase αCeSb_2_, a white+dark two-phase eutectic αCeSb_2_+τ_1_ and a large volume fraction of a grey phase (Sb) which is divorced from the three-phase eutectic αCeSb_2_+τ_1_+(Sb) since (Sb) is the dominant phase near the Sb corner (Fig. 8).

### 2.2 Isothermal section at 823 K over the entire composition range

For the isothermal section at 823 K of the Ce-Fe-Sb ternary system constructed in present work, there exist 15 three-phase regions. In Ref. [1], the detailed descriptions of the three-phase regions containing τ_2_ phase (CeSb)+τ_2_+τ_3_, (CeSb)+τ_2_+αCeSb_2_, (FeSb)+τ_3_+τ_2_, αCeSb_2_+τ_1_+τ_2_ and (FeSb)+τ_1_+τ_2_, are presented. The remaining regions are fully shown in Fig. 9, Fig. 10, Fig. 11, Fig. 12, Fig. 13, Fig. 14, Fig. 15, Fig. 16, Fig. 17, Fig. 18 and described as follows. The equilibrium phase compositions of constituent phases in the heat-treated alloys mentioned below are summarized in Table 6 as presented in Ref. [1].

It should be pointed out that within the XRD spectra of some alloys, the diffraction peaks of Ce_2_O_3_, Sb_2_O_5_ and Fe_2_O_3_ exist since the very easy oxidation of these alloys after their exposing to air, resulting in the dark-grey distributions of cracks and pits.

B1 (γCe)+CeFe_2_+Ce_2_Sb three-phase region *Ce-51Fe-2Sb (#4)* and *Ce-42Fe-6Sb (#5)*

B2 Ce_2_Sb+CeFe_2_+Ce_2_Fe_17_ three-phase region *Ce-60Fe-10Sb (#27)* and *Ce-71Fe-3Sb (#28)*

B3 Ce_2_Sb+ Ce_4_Sb_3_ +Ce_2_Fe_17_ three-phase region *Ce-10Fe-35Sb (#29)*

B4 (CeSb)+Ce_2_Fe_17_+(αFe) three-phase region *Ce-51Fe-23Sb (#30)*

B5 (CeSb)+τ_3_+(αFe) three-phase region

*Ce-35Fe-45Sb(#11), Ce-16Fe-48Sb (#12) and Ce-84Fe-10Sb(#25)*

B6 (FeSb)+τ_3_+(αFe) three-phase region *Ce-72Fe-25Sb(#1)*

B7 αCeSb_2_+τ_1_+Sb three-phase region *Ce-8Fe-82Sb (#14)*

B8 (FeSb)+τ_1_+FeSb_2_ three-phase region *Ce-31Fe-65Sb (#19)*

B9 τ_1_+FeSb_2_+ (Sb) three-phase region *Ce-23.53Fe-70.59Sb (#21)*

B10 τ_3_ + (FeSb) two-phase region *Ce-47Fe-44Sb (#24)*

## Figures and Tables

**Fig. 1:**